# Acute Afterload Mismatch After Transcatheter Tricuspid Valve Repair

**DOI:** 10.1016/j.jaccas.2022.02.008

**Published:** 2022-05-04

**Authors:** Daniel Hagemeyer, Anas Merdad, Geraldine Ong, Neil P. Fam

**Affiliations:** Division of Cardiology, St Michael’s Hospital, University of Toronto, Ontario, Ontario, Canada

**Keywords:** RV dysfunction, structural heart disease, tricuspid regurgitation, transcatheter tricuspid valve repair, TTVr, FAC, fractional area change, LV, left ventricular, NYHA, New York Heart Association, RV, right ventricular, TAPSE, tricuspid annular plane systolic excursion, TEE, transesophageal echocardiogram, TR, tricuspid regurgitation, TTE, transthoracic echocardiogram, TTVI, transcatheter tricuspid valve intervention, TTVr, transcatheter tricuspid valve repair

## Abstract

Acute afterload mismatch and left ventricular dysfunction after mitral valve repair are well established. The impact of transcatheter tricuspid valve repair (TTVr) on right ventricular (RV) function is less clearly defined. To our knowledge, there are no reports of acute RV dysfunction after TTVr. Here we report a case of acute afterload mismatch after successful TTVr. (**Level of Difficulty: Advanced.**)

## Central Illustration

**Figure:**
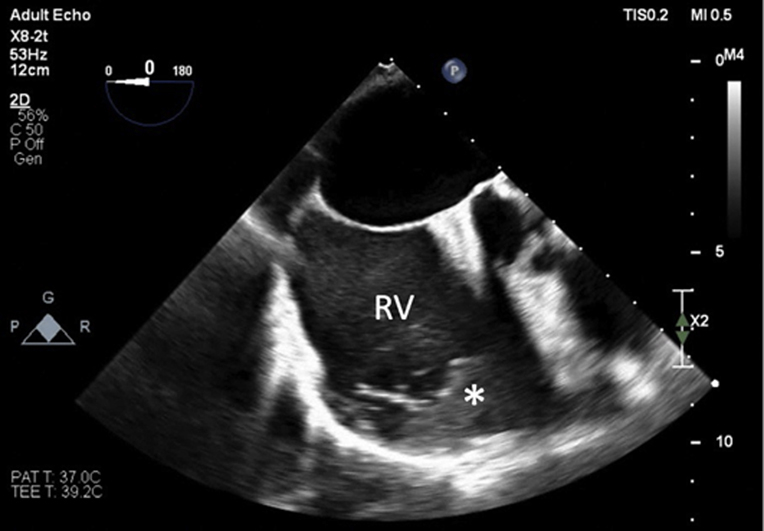

## History of Presentation

An 84-year-old woman presented with symptomatic severe functional tricuspid regurgitation (TR). Guideline-directed medical therapy was initiated, but effective dosing was limited by hypotensive episodes. The patient reported ongoing New York Heart Association (NYHA) functional class III dyspnea, and there was clinical evidence of volume overload.

**Learning Objectives**

• To consider the impact of TTVr on RV afterload and RV systolic function when sudden changes in hemodynamics are observed during the procedure.
• To rapidly consider a differential diagnosis of hypotension during TTVr and be prepared to provide timely hemodynamic support for RV dysfunction.

The transthoracic echocardiogram (TTE) showed normal left ventricular (LV) size and systolic function. The right ventricle was severely dilated with mildly reduced systolic function (tricuspid annular plane systolic excursion [TAPSE], 22 mm; right ventricular [RV] S′ velocity, 11.2 cm/s; and fractional area change [FAC], 40%). Severe functional TR caused by annular dilatation and leaflet tethering was noted. The transesophageal echocardiogram (TEE) demonstrated TR in the anteroseptal commissure extending centrally. Cardiac catheterization revealed patent coronary arteries and elevated filling pressures with mean right atrial pressure of 10 mm Hg and v waves of 20 mm Hg, postcapillary wedge pressure was 19 mm Hg, pulmonary artery pressure was 46/22 mm Hg, mean 31 mm Hg, and LV end-diastolic pressure was 18 mm Hg. The cardiac index was 2.6 L/min (Fick).

The multidisciplinary heart team evaluation deemed the patient a good candidate for transcatheter tricuspid edge-to-edge valve repair. A TriClip XTW (Abbott Cardiovascular) was implanted centrally between the anterior and the septal leaflets, which abolished the TR (Videos 1 and 2). Immediately after the implantation, there was a significant reduction in systemic blood pressure from 125/75 mm Hg to 80/55 mm Hg.

## Past Medical History

Her past medical issues includes heart failure with preserved ejection fraction, atrial fibrillation, and chronic kidney disease.

## Differential Diagnosis

The following causes of acute hypotension were considered: acute occult bleeding, pericardial tamponade, acute RV failure, arrhythmia, acute coronary syndrome, and pulmonary embolism.

## Investigations

**Figure 1:**
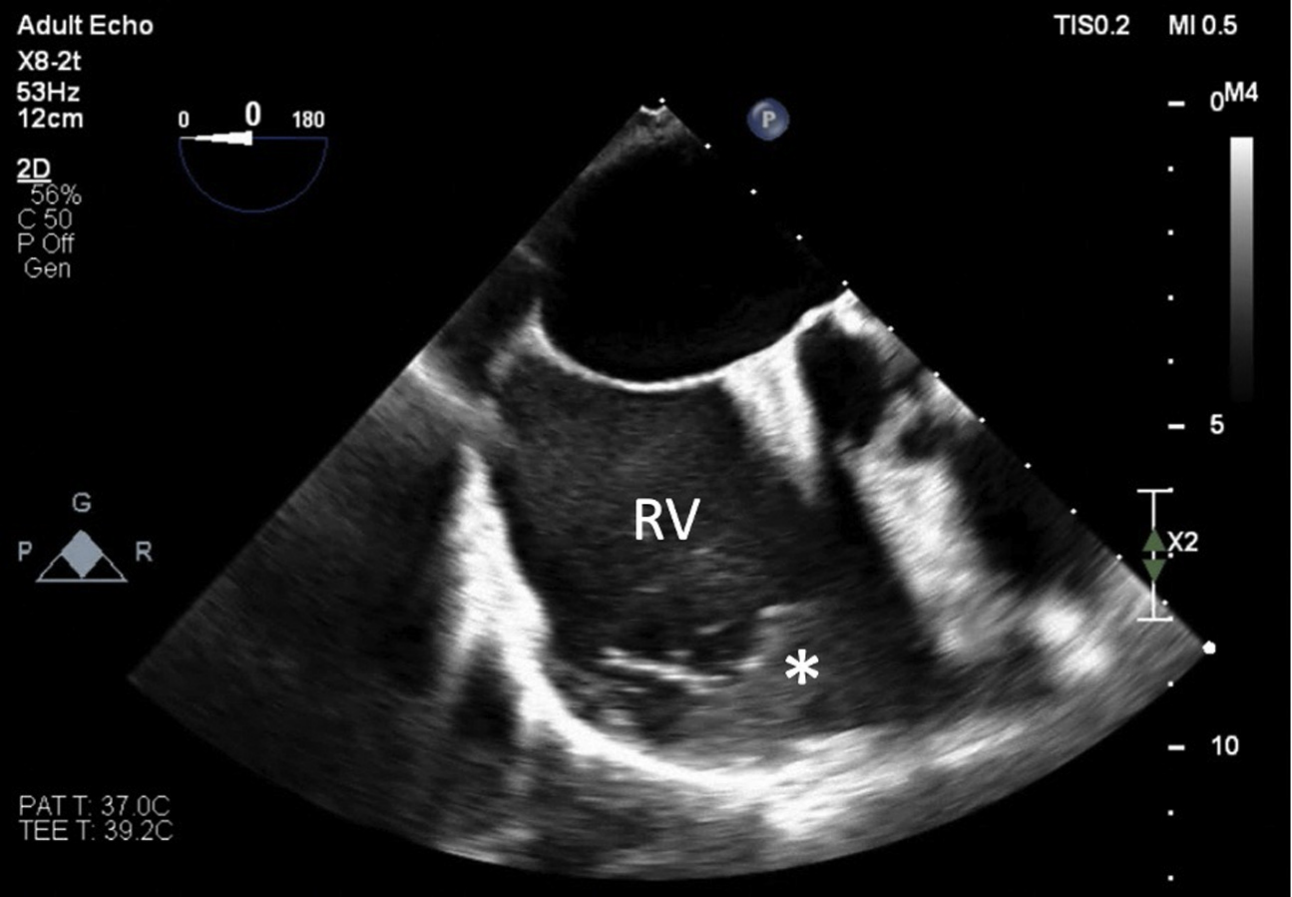
Echocardiographic Imaging Midesophageal echocardiographic view of the right atrium showing sludge or early thrombus **(asterisk)** forming in the right ventricle (RV) after transcatheter tricuspid valve repair.

**Figure 2:**
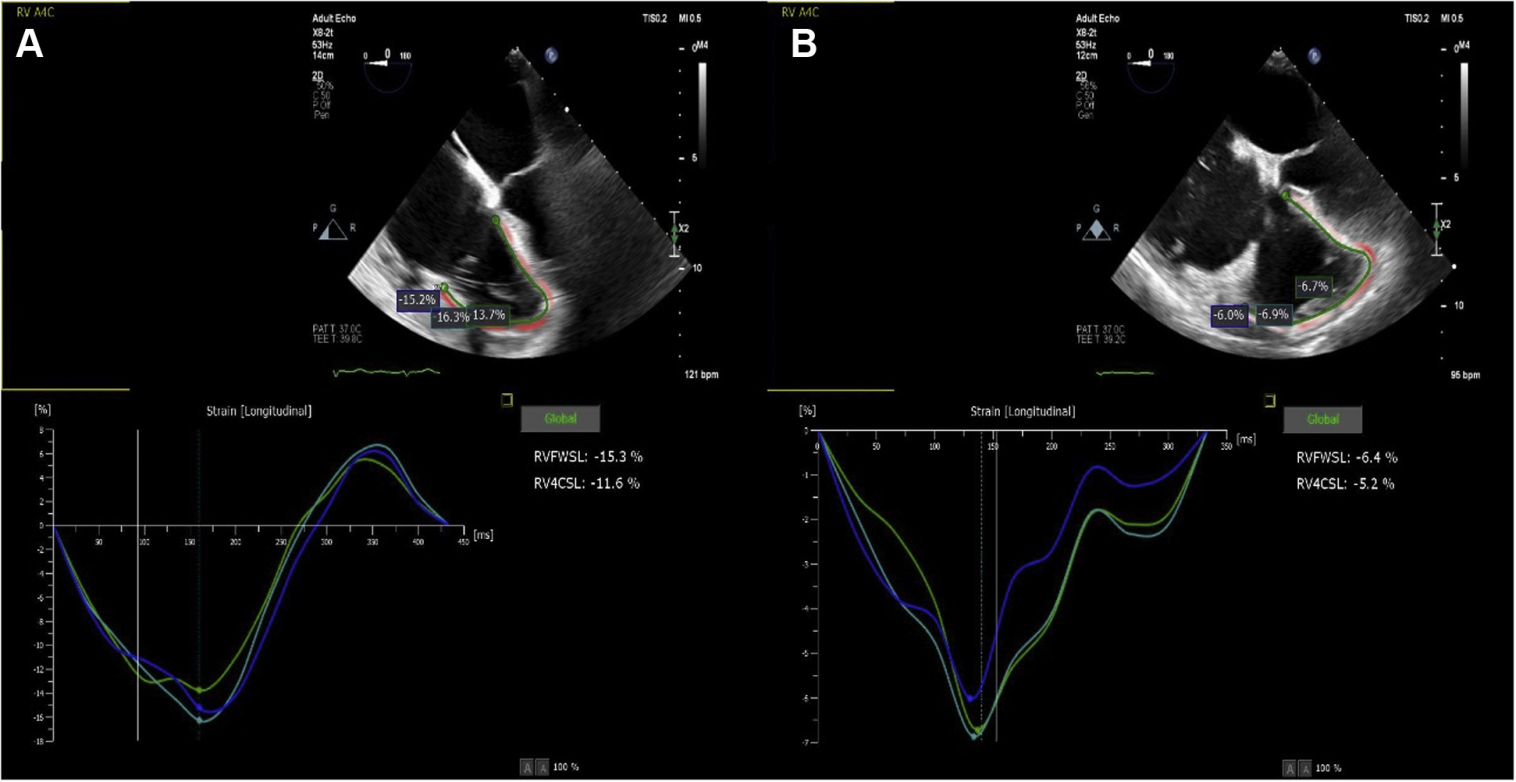
Right Ventricular Longitudinal Strain Measurements The measurements show **(A)** a baseline free-wall strain value of −15.3% and **(B)** a post–transcatheter tricuspid valve repair value of −6.4%. RVFWSL = right ventricular free-wall longitudinal strain; RV4CSL = right ventricular 4-chamber longitudinal strain.

**Figure 3:**
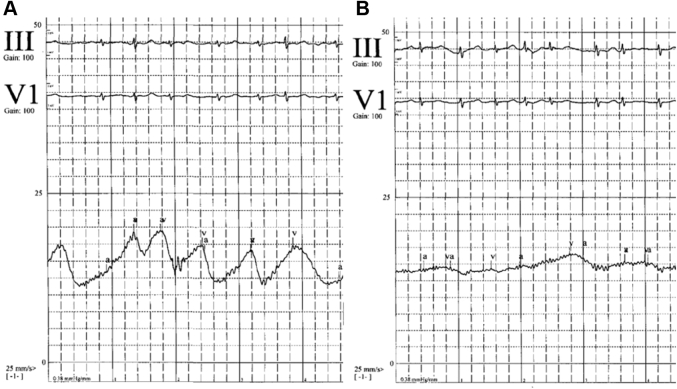
Right Atrial Pressure Tracings The tracings show **(A)** a baseline mean pressure of 14 mm Hg and 20 mm Hg v waves and **(B)** post–transcatheter tricuspid valve repair mean pressure of 13 mm Hg with no significant v wave.

Almost immediately after clip implantation, the TEE demonstrated sudden worsening of the RV function (Videos 3 and 4). In addition, there was apparent spontaneous contrast material “smoke” and sludge seen in the right atrium immediately after TTVr (Video 5, Figure 1). The RV free-wall strain assessment worsened from a baseline of −15.3% to −6.4% (Figures 2A and 2B). The right atrial pressure fell from a mean pressure of 14 mm Hg with v waves of 20 mm Hg to a mean pressure of 13 mm Hg with no v waves (Figures 3A and 3B). Pericardial effusion was ruled out by TEE, with no new wall motion abnormalities, LV dysfunction, or worsening mitral regurgitation. Examination of central access sites, and serial hemoglobin and hematocrit measurements, with persistently elevated right atrial pressure, excluded catastrophic bleeding. No arrhythmias or ischemia were seen on electrocardiographic tracings.

## Management

After cumulative administration of 200 μg of phenylephrine and intravenous saline bolus, the patient’s blood pressure recovered to baseline within 5 minutes, and the RV function improved from severe to moderate dysfunction. A well-seated clip was confirmed on TTE the following day, which demonstrated trace TR and moderately reduced RV systolic function (TAPSE, 11 mm; RV S′ velocity, 6 cm/s; and FAC, 15%). The patient remained normotensive and clinically improved and was discharged home without complications.

## Discussion

The systolic performance of the heart is determined by 3 factors: preload, afterload, and contractility, as described in the Frank-Starling law. Afterload is defined as the total force that opposes sarcomere shortening minus the stretching force that existed before contraction. The stroke volume decreases as the afterload increases. Moreover, a simultaneous increase in end-systolic volume further decreases stroke volume. The implantation of a tricuspid clip leads to a rapid increase in RV afterload because the regurgitant orifice into the low-pressure right atrium is abruptly eliminated. The concept of a decline in LV function caused by afterload mismatch after transcatheter mitral valve repair is well described.^1^, ^2^, ^3^ It has also been shown that an early change in RV function following transcatheter mitral valve repair is predictive of mortality and hospitalization resulting from heart failure during follow-up.^4^

In our case, the increase in afterload resulting from TTVr led to acute, reversible RV dysfunction and hypotension. The impact of RV dysfunction in patients undergoing TTVr is still a matter of debate, and published reports are mainly focused on preinterventional RV function as a predictor of outcome.^5^ We can refer to published surgical reports, which show that preoperative moderate to severe RV dysfunction is a predictor of poor long-term survival in patients undergoing surgical tricuspid valve repair.^6^^,^^7^ Severe RV dysfunction is also associated with worse outcomes after different types of transcatheter tricuspid valve intervention (TTVI).^8^ A recent Trivalve registry analysis suggested that patients with moderate RV dysfunction who underwent TTVI had better survival compared with patients with preserved or severely reduced RV function.^9^ A finding that has implications for patient selection and timing of TTVr. RV contraction patterns can also be characterized by cardiac magnetic resonance imaging, and severe global RV dysfunction was a strong predictor of mortality in patients undergoing TTVr.^10^ Further studies are needed to characterize more precisely the incidence and clinical impact of afterload mismatch after TTVr and the relationship with RV function and long-term outcomes.

## Follow-Up

At 1-month follow-up, the patient’s symptoms have significantly improved. She is enjoying an NYHA functional class I functional capacity, and follow-up TTE demonstrated trace TR and mild RV dysfunction (TAPSE, 15 mm; RV S′ velocity, 7.5 cm/s; and FAC, 22%).

## Conclusions

TTVr may lead to an acute reduction in RV function resulting from abrupt changes in the intracavitary pressure. Operators and anesthesiologists should be aware of this potential complication and be prepared to provide timely hemodynamic support. The long-term clinical impact of afterload mismatch after TTVr remains to be determined.

## Funding Support and Author Disclosures

Dr Hagemeyer has reported financial support from the Gottfried und Julia Bangerter-Rhyner-Stiftung. Dr Fam has consulted for Abbott and Edwards Lifesciences. All other authors have reported that they have no relationships relevant to the contents of this paper to disclose.

## Appendix

Supplemental Video 1 Transgastric Echocardiographic View of the Tricuspid Valve in xPlane Mode With Color Doppler, Demonstrating Significant Tricuspid Regurgitation

Supplemental Video 2 Transgastric Echocardiographic View of the Tricuspid Valve in xPlane Mode With Color Doppler, Demonstrating Trace Residual Tricuspid Regurgitation Post-TTVr

Supplemental Video 3 Midesophageal Echocardiographic View of the Right Ventricle Showing Baseline Mildly Reduced Systolic Function

Supplemental Video 4 Midesophageal Echocardiographic View of the Right Ventricle Showing Severely Reduced Systolic Function After TTVr

Supplemental Video 5 Significant Stasis With Spontaneous Contrast “Smoke” and Sludge Seen in the Right Atrium Immediately After TTVr

## References

[1] Melisurgo G., Ajello S., Pappalardo F. (2014). Afterload mismatch after MitraClip insertion for functional mitral regurgitation. Am J Cardiol.

[2] Hagnäs M.J., Grasso C., Di Salvo M.E. (2021). Impact of post-procedural change in left ventricle systolic function on survival after percutaneous edge-to-edge mitral valve repair. J Clin Med.

[3] Jogani S., Van de Heyning C.M., Paelinck B.P. (2020). Afterload mismatch after MitraClip implantation: intraoperative assessment and prognostic implications. J Invasive Cardiol.

[4] Sugiura A., Shamekhi J., Goto T. (Published online October 20, 2021). Early response of right-ventricular function to percutaneous mitral valve repair. Clin Res Cardiol.

[5] Preda A., Melillo F., Liberale L., Montecucco F., Agricola E. (2021). Right ventricle dysfunction assessment for transcatheter tricuspid valve repair: a matter of debate. Eur J Clin Invest.

[6] Algarni K.D., Arafat A., Algarni A.D. (2021). Degree of right ventricular dysfunction dictates outcomes after tricuspid valve repair concomitant with left-side valve surgery. Gen Thorac Cardiovasc Surg.

[7] Subbotina I., Girdauskas E., Bernhardt A.M., Sinning C., Reichenspurner H., Sill B. (2017). Comparison of outcomes of tricuspid valve surgery in patients with reduced and normal right ventricular function. Thorac Cardiovasc Surg.

[8] Miura M., Alessandrini H., Alkhodair A. (2020). Impact of massive or torrential tricuspid regurgitation in patients undergoing transcatheter tricuspid valve intervention. J Am Coll Cardiol Intv.

[9] Schlotter F., Miura M., Kresoja K.P. (2021). Outcomes of transcatheter tricuspid valve intervention by right ventricular function: a multicentre propensity-matched analysis. EuroIntervention.

[10] Kresoja K.P., Rommel K.P., Lücke C. (2021). Right ventricular contraction patterns in patients undergoing transcatheter tricuspid valve repair for severe tricuspid regurgitation. J Am Coll Cardiol Intv.

